# Underdiagnosis bias of artificial intelligence algorithms applied to chest radiographs in under-served patient populations

**DOI:** 10.1038/s41591-021-01595-0

**Published:** 2021-12-10

**Authors:** Laleh Seyyed-Kalantari, Haoran Zhang, Matthew B. A. McDermott, Irene Y. Chen, Marzyeh Ghassemi

**Affiliations:** 1 University of Toronto, Toronto, Ontario Canada; 2 Vector Institute, Toronto, Ontario Canada; 3 Massachusetts Institute of Technology, Cambridge, MA USA

**Keywords:** Machine learning, Medical imaging

## Abstract

Artificial intelligence (AI) systems have increasingly achieved expert-level performance in medical imaging applications. However, there is growing concern that such AI systems may reflect and amplify human bias, and reduce the quality of their performance in historically under-served populations such as female patients, Black patients, or patients of low socioeconomic status. Such biases are especially troubling in the context of underdiagnosis, whereby the AI algorithm would inaccurately label an individual with a disease as healthy, potentially delaying access to care. Here, we examine algorithmic underdiagnosis in chest X-ray pathology classification across three large chest X-ray datasets, as well as one multi-source dataset. We find that classifiers produced using state-of-the-art computer vision techniques consistently and selectively underdiagnosed under-served patient populations and that the underdiagnosis rate was higher for intersectional under-served subpopulations, for example, Hispanic female patients. Deployment of AI systems using medical imaging for disease diagnosis with such biases risks exacerbation of existing care biases and can potentially lead to unequal access to medical treatment, thereby raising ethical concerns for the use of these models in the clinic.

## Abstract (web summary)

Artificial intelligence algorithms trained using chest X-rays consistently underdiagnose pulmonary abnormalities or diseases in historically under-served patient populations, raising ethical concerns about the clinical use of such algorithms.

## Main

As artificial intelligence (AI) algorithms increasingly affect decision-making in society^1^, researchers have raised concerns about algorithms creating or amplifying biases^2–11^. In this work we define biases as differences in performance against, or in favor of, a subpopulation for a predictive task (for example, different performance on disease diagnosis in Black compared with white patients). Although AI algorithms in specific circumstances can potentially reduce bias^12^, direct application of AI has also been shown to systematize biases in a range of settings^2–7,13,14^. This tension is particularly pressing in healthcare, where AI systems could improve patient health^4^ but can also exhibit biases^2–7^. Motivated by the global radiologist shortage^15^ as well as by demonstrations that AI algorithms can match specialist performance particularly in medical imaging^16^, AI-based diagnostic tools present a clear incentive for real-world deployment.

Although much work has been done in algorithmic bias^13^ and bias in health^2–11^, the topic of AI-driven underdiagnosis has been relatively unexplored. Crucially, underdiagnosis, defined as falsely claiming that the patient is healthy, leads to no clinical treatment when a patient needs it most, and could be harmful in radiology specifically^17,18^. Given that automatic screening tools are actively being developed in research^19–23^ and have been shown to match specialist performance^16^, underdiagnosis in AI-based diagnostic algorithms can be a crucial concern if used in the clinical pipeline for patient triage. Triage is an important diagnostic first step in which patients who are falsely diagnosed as healthy are given lower priority for a clinician visit. As a result, the patient will not receive much-needed attention in a timely manner. Underdiagnosis is potentially worse than misdiagnosis, because in the latter case, the patient still receives clinical care, and the clinician can use other symptoms and data sources to clarify the mistake. Initial results have demonstrated that AI can reduce underdiagnosis in general^24,25^ but these studies do not deeply consider the existing clinical biases in underdiagnosis against under-served subpopulations. For example, Black patients tend to be more underdiagnosed in chronic obstructive pulmonary disease than non-Hispanic white patients^9^.

**Fig. 1:**
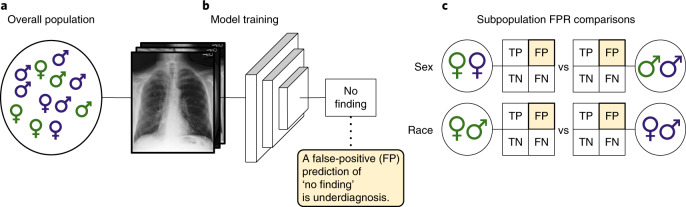
The model pipeline. **a**, We examine chest radiographs across several datasets with diverse populations. **b**, A deep learning model is then trained from these data (training across all patients simultaneously) to predict the presence of the no finding label, which indicates that the algorithm did not detect disease for the image. **c**, The underdiagnosis rate (that is, the false-positive rate (FP) of the no finding label) of this model is then compared in different subpopulations (including sex, race/ethnicity, age and insurance type) to examine the algorithm’s underdiagnosis rate. FN, false negative; TN, true negative; TP, true positive. Symbol colors indicate different races of male and female patients.

Here, we perform a systematic study of underdiagnosis bias in the AI-based chest X-ray (CXR) prediction models, designed to predict diagnostic labels from X-ray images, in three large public radiology datasets, MIMIC-CXR (CXR)^26^, CheXpert (CXP)^27^ and ChestX-ray14 (US National Institutes of Health (NIH))^28^, as well as a multi-source dataset combining all three on shared diseases. We focus our underdiagnosis study on individual and intersectional subgroups spanning race, socioeconomic status (as assessed via the proxy of insurance type), sex and age. The choice of these subgroups is motivated by the clear history, in both traditional medicine and AI algorithms, of bias for subgroups on these axes^6,8,10,11^. An illustration of our model pipeline is presented in Fig. 1.

## Results

A standard practice among the AI-based medical image classifiers is to train a model and report the model performance on the overall population regardless of the patient membership to subpopulations^16,19–23^. Motivated by known differences in disease manifestation in patients by sex^6^, age^29^, race/ethnicity^8^ and the effect of insurance type in quality of received care^11^, we report results for all of these factors. We use insurance type as an imperfect proxy of socioeconomic status because, for example, patients with Medicaid insurance are often in the low income bracket. Given that binarized predictions are often required for clinical decision-making at the individual level, we define and quantify the underdiagnosis rate based on the binarized model predictions. To assess model decision biases in underdiagnosed patients, we compare underdiagnosis rates across subpopulations in the overall population. We define the underdiagnosis rate as the false-positive rate (FPR) of the binarized model prediction for the ‘no finding’ label, indicating that no disease is diagnosed, at the levels of subgroup (for example, female) and intersectional identities (for example, Black and female).

We measure the underdiagnosis rate in distinct chest X-ray diagnosis models trained in four dataset settings: MIMIC-CXR (CXR, 371,858 images from 65,079 patients)^26^, CheXpert (CXP, 223,648 images from 64,740 patients)^27^, ChestX-ray14 (NIH, 112,120 images from 30,805 patients)^28^, and a multi-source combination of all three (ALL, 707,626 images from 129,819 patients) on shared labels. The CXR, CXP and NIH datasets have relatively equal rates of male and female patients, and most patients are between 40 and 80 years old. Note that the CXP and NIH datasets report only patient sex and age, whereas the CXR dataset additionally reports patient race/ethnicity and insurance type for a large subset of images. In the CXR dataset we note that both race/ethnicity and insurance type are highly skewed. We use the term ‘sex’ to match the reported terminology in the underlying data. Gender presentation plays a large role in societal biases but these data are not routinely collected^26–28^. More detailed summary statistics for the datasets are listed in Table 1. The full data collection description per dataset is available in the Methods.

**Table 1:** Summary statistics for all datasets

Subgroup	Attribute	CXR	CXP	NIH	ALL
	No. of images	371,858	223,648	112,120	707,626
**Sex (%)**	Male	52.17	59.36	56.49	55.13
	Female	47.83	40.64	43.51	44.87
**Age (%)**	0–20 years	2.20	0.87	6.09	2.40
	20–40 years	19.51	13.18	25.96	18.53
	40–60 years	37.20	31.00	43.83	36.29
	60–80 years	34.12	38.94	23.11	33.90
	>80 years	6.96	16.01	1.01	8.88
**Race/Ethnicity (%)**	Asian	3.24	–	–	–
	Black	18.59	–	–	–
	Hispanic	6.41	–	–	–
	Native	0.29	–	–	–
	White	67.64	–	–	–
	Other	3.83	–	–	–
**Insurance (%)**	Medicare	46.07	–	–	–
	Medicaid	8.98	–	–	–
	Other	44.95	–	–	–
	AUC ± 95% CI	0.834 ± 0.001	0.805 ± 0.001	0.835 ± 0.002	0.859 ± 0.001

The datasets studied are MIMIC-CXR (CXR)^26^, CheXpert (CXP)^27^, ChestX-ray14 (NIH)^28^ and a multi-source dataset (ALL) composed of aggregated data from the CXR, CXP and NIH datasets using the shared labels (disease labels and the no finding label) in all three datasets. The deep learning model is trained on each of the CXR, CXP, NIH and ALL datasets. The model’s AUCs are then estimated for each of the labels in the CXR (14 labels), CXP (14 labels), NIH (15 labels) and ALL (8 labels) datasets, and are averaged over all of the labels for each dataset. The reported AUC ± 95% confidence interval (CI) for each dataset is then the average of the AUCs for the five trained models with different random seeds using the same train–validation–test split.

### Underdiagnosis in under-served patient subpopulations

**Fig. 2:**
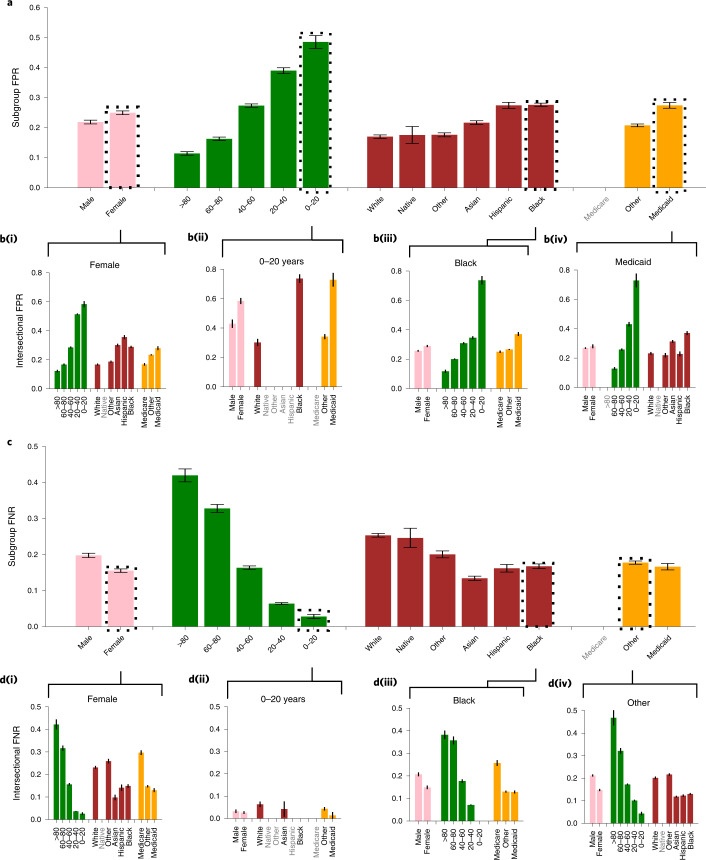
Analysis of underdiagnosis across subgroups of sex, age, race/ethnicity and insurance type in the MIMIC-CXR (CXR) dataset. **a**, The underdiagnosis rate, as measured by the no finding FPR, in the indicated patient subpopulations. **b**, Intersectional underdiagnosis rates for female patients (**b(i)**), patients aged 0–20 years (**b(ii)**), Black patients (**b(iii)**), and patients with Medicaid (**b(iv)**). **c**,**d**, The overdiagnosis rate, as measured by the no finding FNR in the same patient subpopulations as in **a** and **b**. The results are averaged over five trained models with different random seeds on the same train–validation–test splits. 95% confidence intervals are shown. Subgroups with too few members to be studied reliably (≤15) are labeled in gray text and the results for these subgroups are omitted. Data for the Medicare subgroup are also omitted, given that data for this subgroup are highly confounded by patient age.

**Extended Data Fig. 1:**
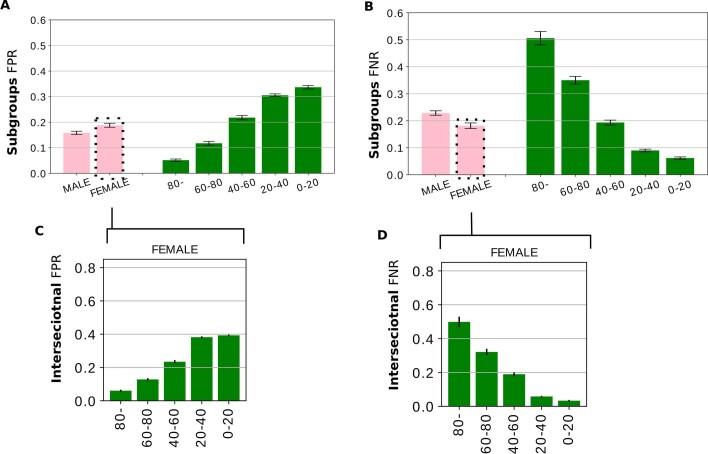
Analyzing underdiagnoses over subgroups of sex, age, within ALL dataset (combined CXR, CXP and NIH dataset on shared labels). **Fig. S1**. Analyzing underdiagnoses over subgroups of sex, age, within ALL dataset (combined CXR, CXP and NIH dataset on shared labels). The results are averaged over 5 trained model with different random seed ± 95% confidence interval (CI). **A**. The underdiagnosis rate (measured by ‘No Finding’ FPR). **B**. The overdiagnosis rate (‘No Finding’ False Negative Rate (FNR)) over subgroups of sex, age. **C**. The intersectional underdiagnosis rates within only female patients. **D**. Examining the overdiagnosis rate for the intersectional identities. The number of images with actual 0 or 1 ‘No Finding’ label in the age - sex intersections in the test dataset is presented in Supplementary Table 1.

**Extended Data Fig. 2:**
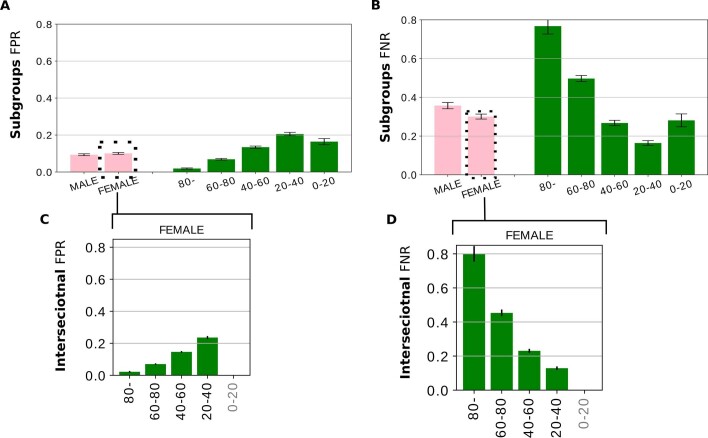
Analyzing underdiagnoses over subgroups of sex, age, within CheXpert (CXP) dataset. **Fig. S2**. Analyzing underdiagnoses over subgroups of sex, age, within CheXpert (CXP) dataset. The results are averaged over 5 trained model with different random seed ± 95% CI. **A**. The underdiagnosis rate is FPR in ‘No Finding’. **B**. Examining the overdiagnosis rate (‘No Finding’ FNR) over sex and age subgroups, **C**. The intersectional underdiagnosis rates within only female patients, and **D**. measure the overdiagnosis rate for the intersectional identities. The subgroups labeled in gray text, with results omitted, indicate the subgroup has too few members (<= 15) to be used reliably. The number of images with actual 0 or 1 ‘No Finding’ label in the age - sex intersections in the test dataset is presented in Supplementary Table 1.

**Extended Data Fig. 3:**
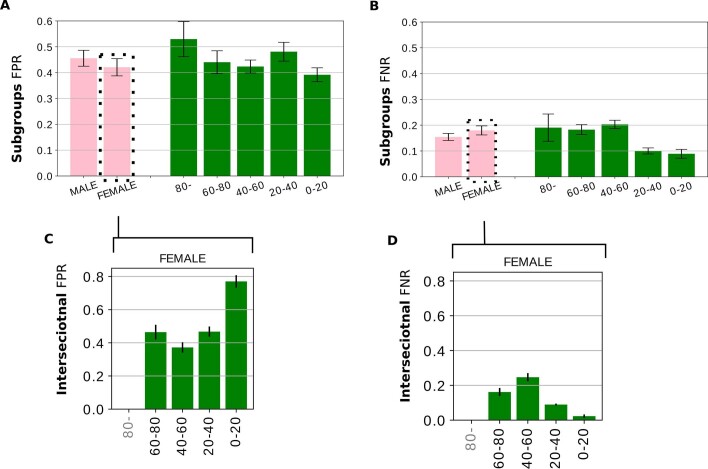
Analyzing underdiagnoses over subgroups of sex, age, within ChestX-ray14 (NIH) dataset. **Fig. S3**. Analyzing underdiagnoses over subgroups of sex, age, within ChestX-ray14 (NIH) dataset. The results are averaged over 5 trained model with different random seed ± 95% confidence interval (CI). **A**. The underdiagnosis rate (‘No Finding’ FPR). **B**. The over diagnosis rate (‘No Finding’ FNR) over subgroups of sex and age. **C**. The intersectional underdiagnosis rates within only female patients. **D**. The over diagnosis rate for the intersectional identities. The subgroups labeled in gray text, with results omitted, indicate the subgroup has too few members (<= 15) to be used reliably. The number of images with actual 0 or 1 ‘No Finding’ label in the age - sex intersections in the test dataset is presented in Supplementary Table 1.

We find that the underdiagnosis rate for all datasets differs in all considered subpopulations. In Fig. 2a we show the subgroup-specific underdiagnosis for CXR dataset on race/ethnicity, sex, age and insurance type. We observed that female patients, patients under 20 years old, Black patients, Hispanic patients and patients with Medicaid insurance receive higher rates of algorithmic underdiagnosis than other groups. In other words, these groups are at a higher risk of being falsely flagged as healthy, and of receiving no clinical treatment. We summarize a similar analysis of the other datasets (CXP, NIH and ALL) in Table 2 and Extended Data Figs 1–3. Additional data for image counts on the test set per subgroup are given in Supplementary Tables 1–3.

**Table 2:** Age and sex subgroups with the most underdiagnosis and least overdiagnosis for all four datasets

Subpopulation	CXR	CXP	NIH	ALL
**Most underdiagnosed group**				
**Sex**	Female	Female	Male	Female
**Age (years)**	0–20	20–40	>80	0–20
**Female–Age (years)**	0–20	20–40	0–20	0–20
**Least overdiagnosed group**				
**Sex**	Female	Female	Male	Female
**Age (years)**	0–20	20–40	0–20	0–20
**Female–Age (years)**	0–20	20–40	0–20	0–20

We find that the patterns of bias are consistent across the CXR (Fig. 2a), ALL (Extended Data Fig. 1a) and CXP (Extended Data Fig. 2a) datasets—that is, female and younger patients have the largest underdiagnosis rates. However, in the NIH dataset (Extended Data Fig. 3a), male patients and patients aged >80 years have the largest underdiagnosis rate. This may be partially due to the small subset sizes, given that the test set for patients aged >80 years has only 37 samples with the no finding label with which to estimate FPR. The NIH dataset is also different from the CXP and CXR datasets in several key ways: it contains frontal images only, rather than frontal and lateral images; it does not use the CheXpert labelers^27^ to create diagnostic labels; and it has only seven of the shared disease labels instead of 14, meaning that the no finding label denotes the absence of different diseases. Moreover, the NIH dataset originates from a hospital that “...does not routinely provide standard diagnostic and treatment services. Admission is selective: patients are chosen by Institute physicians solely because they have an illness being studied by those Institutes.” (from https://clinicalcenter.nih.gov/about/welcome/faq.html). Thus, the NIH dataset may have less diverse samples than the CXP and CXR datasets, which originate from clinical hospitals (see Methods for more detail).

### Underdiagnosis in intersectional groups

We investigate intersectional groups, here defined as patients who belong to two subpopulations, for example, Black female patients. Similar to prior work in facial detection^14^, we find that intersectional subgroups (Fig. 2b) often have compounded biases in algorithmic underdiagnosis. For instance, in the CXR dataset, Hispanic female patients have a higher underdiagnosis rate—that is, a no finding FPR—than white female patients (Fig. 2b(i)). Also, the intersectional subgroups of patients who are aged 0–20 years and female, aged 0–20 years and Black, and aged 0–20 years with Medicaid insurance have the largest underdiagnosis rates (Fig. 2b(ii)). The underdiagnosis rate for the intersection of Black patients with another subgroup of age, sex and insurance type (Fig. 2b(iii)) and that for patients with Medicaid insurance with another subgroup of sex, age and race/ethnicity (Fig. 2b(iv)) is also shown in Fig. 2b. We observe that patients who belong to two under-served subgroups have a larger underdiagnosis rate. In other words, not all female patients are misdiagnosed at the same rate (for example, Hispanic female patients are misdiagnosed more than white female patients) (Fig. 2b(i)). The intersectional underdiagnosis rate for the ALL, CXP and NIH datasets is shown in Extended Data Figs. 1c, 2c and 3c, respectively, where the intersectional identities are often underdiagnosed even more heavily than the group in aggregate. The most underdiagnosed age groups for female patients are listed under the Female–Age attribute in Table 2.

### Underdiagnosis or overall noise

The false-negative rate (FNR) for no finding (Fig. 2c) and FPR (Fig. 2a) show an inverse relationship across different under-served subgroups in the CXR dataset. Such an inverse relationship also exists for intersectional subgroups (Fig. 2d). This finding is consistent across all datasets (compare both the overall and intersectional FPR and FNR in Extended Data Figs. 1–3), except for the age >80 years and 0–20 years subgroups in the NIH dataset, which may again be due to the small number of samples in the >80 years subgroups or to potential dataset selection bias (Methods). The fact that FPR and FNR show an inverse relationship, rather than an increase for both FPR and FNR, suggests that under-served subpopulations are being aggressively flagged erroneously as healthy by the algorithm, without a corresponding increase of instances of erroneous diagnoses of disease by the algorithm. This is consistent only with selective algorithmic underdiagnosis rather than simple, undirected errors that could arise from a higher rate of noise alone. Using Fig. 2c,d and Extended Data Figs. 1b,d,2b,d,3b,d we summarize subpopulations with the lowest overdiagnosis rates (lowest FNR for no finding) across the datasets in Table 2.

### Likelihood of underdiagnosis in specific diseases

The distribution of disease prevalence in the underdiagnosed patient population is significantly different to that in the general patient population. We compare the disease prevalence in the unhealthy population and the underdiagnosed population for the intersections of race/ethnicity and sex in Supplementary Table 4. For example, underdiagnosed populations are proportionally more likely to have a positive label for lung lesion and less likely to have a positive label for pleural effusion. This suggests that the task of disease detection is more difficult for some diseases than others.

### Fairness definitions in a healthcare context

Our study considers underdiagnosis as the main fairness concern, due to its potentially harmful impact on patients, such as causing a delay in receiving treatment (for example, assigning lower priority to the underdiagnosed population in a triage use case). We acknowledge that depending on the use case of the algorithm there are many other fairness definitions one may consider. One such definition is predictive parity, which implies equal positive predictive value, or, equivalently, false discovery rate (FDR) between the groups^30^. In Supplementary Table 6 we report the additional data for FDR of a no disease diagnosis (that is, the likelihood that the patient is ill given that the classifier predicts no finding). We observe that, similar to FPR and FNR, significant gaps exist across many protected attributes. In particular, these disparities tend to follow a different pattern of that seen for FPR, favoring, for example, female people over male people and younger people over older people. The underlying cause is the difference in prevalence between groups—that is, given that there are far fewer sick people in the 0–20 year age group (Supplementary Tables 1–3), we will have relatively fewer false positives and true negatives, which, keeping all else constant, will cause a decrease in the FDR.

## Discussion

We have shown consistent underdiagnosis in three large, public datasets in the chest X-ray domain. The algorithms trained on all settings exhibit systematic underdiagnosis biases in under-served subpopulations, such as female patients, Black patients, Hispanic patients, younger patients and patients of lower socioeconomic status (with Medicaid insurance). We found that these effects persist for intersectional subgroups (for example, Black female patients) but are not consistently worse in the smallest intersectional groups. The specific subpopulations most affected vary in the NIH dataset, specifically male patients and patients aged >80 years, which should be explored further. Beyond these immediate take-aways, there are several topics for further discussion and investigation.

First, we highlight that automatic labeling from notes should be carefully audited. We note that in chest X-ray datasets, there has been a general shift in machine learning from manual image labeling to automatic labeling, with natural language processing (NLP)-based methods used to generate the labels in radiology reports. This has resulted in large annotated chest X-ray datasets^26–28^ that are widely used for training deep learning models and for providing AI solutions^16,19–23,31^. Although automatic labelers have been validated for labeling quality^26–28^ and adapted as reliable ground truth, the performance of these labelers in different subpopulations has not been explored. Given that NLP-based techniques have shown biases against under-represented subpopulations in both medical^32^ and non-medical^33^ domains, the automatic labeler could potentially be a large source of bias.

Second, bias amplification is likely to be generalizable. The present results should be considered in the context of known biases in clinical care itself, in which under-served subpopulations are often underdiagnosed by doctors without a simultaneous increase in privileged group overdiagnosis^9^. Our prediction labels are extracted from clinical records, and are therefore not an unbiased ground truth; in other words, our labels may already contain the same bias that our model is then additionally demonstrating. This is a form of bias amplification, when a model’s predicted outputs amplify a known source of error in the process of data generation^34^ or data distribution^35^. This is an especially dangerous outcome for machine learning models in healthcare, given that existing biases in health practice risk being magnified, rather than ameliorated, by algorithmic decisions based on large (707,626 images), multi-source datasets.

We note that some of our observed differences in underdiagnosis have been established in other areas in clinical care, such as underdiagnosis of female patients^9,10^, Black patients^5,8,9^ and patients with a low socioeconomic status^36^. Therefore, we would expect our results to hold regardless of the algorithm used, given that the disparities probably originate from the data. Moreover, missing data, small sample size and the consistently suboptimal care delivered to some subpopulations have been sources of bias amplification concerns^36^. Patients with low socioeconomic status may have fewer interactions with the healthcare system, or they may be more likely to visit a teaching or research clinic where clinical reasoning or treatment plans may be different^36^. Our results may not be replicable in health settings in which the dynamics of sex or racial identity are different, or in which the health insurance system operates differently.

Third, although there are possible post-hoc technical solutions for imposing fairness, it comes with deep flaws. One simple post-processing method for achieving equal FNR and FPR across subgroups is the selection of different thresholds for different groups corresponding to the intersection of their receiver operating characteristic (ROC) curves^37,38^. However, there are many flaws involved in using a different threshold for each group. For example, for intersectional subgroups with small populations, an accurate approximation of the threshold might be difficult to obtain because of the large degree of uncertainty. The number of thresholds required to be computed also grows exponentially with the number of protected attributes, which makes it largely infeasible for intersections of three or more protected attributes. Additionally, race and ethnicity are partially social constructs, with unclear boundaries. As a result, self-reported race and ethnicity may be inconsistent and may vary based on individual factors such as age, socioeconomic level or the level of acculturation to society^39^. This heterogeneity in self-identification may result in lower model performance for patients of groups in which self-identification criteria are more complex. Finally, this solution is ideal only in cases in which the per-group ROC curves have intersections. In cases in which the ROC curves do not intersect, or we desire an FNR–FPR combination not corresponding to an intersection between curves, achieving equal FNR and FPR would require randomization—that is, systematic worsening of the model performance in particular subgroups^37^. It is unclear whether worsening the overall model performance for one subgroup to achieve equality is ethically desirable. This is especially relevant in the medical context, in which we do not expect that all subgroups would have similar areas under the ROC curve (AUCs), given that the difficulty of the problem often varies with the protected group, for example, with age. We do note that equal FPR alone is easily achievable through threshold adjustments if the underdiagnosis is the main fairness concern. However, such a solution could still induce large overdiagnosis (FNR) disparities, in addition to requiring knowledge of the patients’ group membership.

Fourth, despite the fact that we do not have the same disease prevalence between subgroups based on real data^26–28^, and our choice of fairness metrics does not directly involve prevalence between subgroups, we stress that equal underdiagnosis rates between subgroups of age, sex and race/ethnicity are still desired. If a classifier deployed in a clinical pipeline mistakenly underdiagnosed a certain subgroup (for example, Black patients) more than others due to the lower prevalence of the disease, this still leads to disadvantage for members of that group and could lead to serious ethical concerns^8^.

Fifth, we note that fairness definitions must be chosen carefully in a healthcare context, given that many definitions are not concurrently satisfiable as shown through fairness impossibility theorems^38,40^. For example, given that the base rates of the two groups are different, it is impossible for them to have equal FNR, FPR and FDR, unless the classifier predicts all samples perfectly^40^.

Last, regulatory and policy decision-makers must consider underdiagnosis. Our work demonstrates the importance of detailed evaluation of medical algorithms, even those that are built with seemingly robust model pipelines. Given that medical algorithms are increasingly widespread, practitioners should assess key metrics such as differences in underdiagnosis rates and other health disparities during the model development process and again after deployment. Furthermore, the clinical application and historical context of each medical algorithm and the potential biases in data gathering should guide the type and frequency of introspection. Moving AI-based decision-making models from paper to practice without considering the biases that we have shown, as well as the ability of AI-based models to detect attributes such as the race of the patients from X-rays^41^, may harm under-served patients. We therefore suggest fairness checks, for underdiagnosis to be merged into the regulatory approval of medical decision-making algorithms before deployment, particularly in the case of triage, where underdiagnosis delays access to care. Moreover, developers, practitioners and the clinical staff need to take into account biases such as the underdiagnosis of under-served populations in the AI-based medical decision-making algorithms and its harmful effect^17,18^ on patients. Additionally, given that different fairness metrics are not concurrently satisfiable, a thorough use-based study to analyze the advantages and disadvantages of different fairness metrics is essential. Such studies guide policymakers to standardize the fairness checks of AI-based diagnostic algorithms prior to deployment. Finally, it is important to acknowledge that a rapidly changing research landscape can yield iterative modifications to regulations as we continue to better understand how algorithmic bias can permeate medical algorithms.

In conclusion, we demonstrate evidence of AI-based underdiagnosis against under-served subpopulations in diagnostic algorithms trained on chest X-rays. Clinically, underdiagnosis is of key importance because undiagnosed patients incorrectly receive no treatment. We observe, across three large-scale datasets and a combined multi-source dataset, which under-served subpopulations are consistently at significant risk of algorithmic underdiagnosis. Additionally, patients in intersectional subgroups (for example, Black female patients) are particularly susceptible to algorithmic underdiagnosis. Our findings demonstrate a concrete way that deployed algorithms (for example, https://models.acrdsi.org/) could escalate existing systemic health inequities if there is not a robust audit of performance disparities across subpopulations. As algorithms move from the laboratory to the real world, we must consider the ethical concerns regarding the accessibility of medical treatment for under-served subpopulations and the effective and ethical deployment of these models.

## Methods

### Dataset

We have utilized three large public chest X-ray datasets in this study: MIMIC-CXR (CXR)^26^, CheXpert (CXP)^27^ and ChestX-ray14 (NIH)^28^. The CXR dataset was collected from Beth Israel Deaconess Medical Center (Boston, MA, United States) between 2011 and 2016, the CXP dataset was collected from Stanford Hospital (Stanford, CA, United States) between October 2002 and July 2017, and the NIH dataset was collected from the NIH Clinical Center (Bethesda, MD, United States) between 1992 and 2015. The CXR and CXP datasets contain 14 diagnosis labels and the NIH dataset contains 15 diagnosis labels, and all contain one extra label indicating no predicted diagnosis of the other disease labels (‘no finding’). We focus on the no finding label for our underdiagnosis analysis. Disease labels are consistent in CXR and CXP, while only eight labels of the NIH dataset are matched with them. In the multi-source ALL dataset we aggregate the three aforementioned datasets on the eight shared labels.

### Dataset collection and inclusion criteria

Because of the size of these large datasets and the fact that no exclusion criteria are mentioned in the dataset descriptions, we do not anticipate any issues with selection bias and assume that the collected datasets are representative of patients at these hospitals over the specified years. Only the ChestX-ray14 dataset is gathered from the NIH clinical research dedicated hospital, where patients are treated without charge and are selected based on whether the illness is being studied by the Institutes.

The NIH dataset has only frontal view images, whereas the other datasets have both frontal and lateral view images. We include all of the images of each dataset, regardless of the view, in the model training and evaluation. The race/ethnicity and sex data are self-reported in the MIMIC-CXR dataset and age is reported at a patient’s first admission. In the CheXpert dataset, sex is assigned by clinicians and the age is reported at the time of the examination. In the ChestX-ray14 dataset, the sex is self-identified and the age corresponds to the time of the examination. In the MIMIC-CXR dataset, the race/ethnicity and insurance type data were collected only if the patient was admitted to an intensive care unit, therefore there are around ~100,000 X-rays for which we do not have these data (these are X-rays done for patients who were admitted only to the emergency department. The reported race/ethnicity in the MIMIC-CXR dataset are white, other, Hispanic/Latino, Black/African American, and American Indian/Alaska Native, and in this study we have used the shorter terminology white, other, Hispanic, Black, and Native for each group, respectively.

### Definition and quantification of the fairness metrics

Commonly used fairness definitions such as equality of odds and equality of opportunity^37^ rely on equal binarized prediction metrics across subgroups. We evaluate the fairness of models in binarized fairness metrics because binarized prediction is most often required for clinical decision-making at the individual level. To assess model decision biases in underdiagnosed patients we compare underdiagnosis rates across subpopulations. We define the underdiagnosis rate as the FPR of the binarized model prediction for the no finding label at the levels of the subgroup (*s*_*j*_), that is, ${\mathrm{FPR}}_{s_j}$ (for example, female patients) and the intersectional (*s*_*i,j*_) identities, that is, ${\mathrm{FPR}}_{s_{i,j}}$ (for example, Black female patients), as given by:

1 $${\mathrm{FPR}}_{s_j} = P\left[ {\hat Y = 1|s_j,\,Y = 0} \right]$$

2 $${\mathrm{FPR}}_{s_{i,j}} = P\left[ {\hat Y = 1|s_{i,j},\,Y = 0} \right]$$

where *i*, *j* denote subgroups with distinct attributes, *Y* is the true label and $\hat Y$ is the predicted label. We then compare these underdiagnosis rates across subpopulations including age and sex in all four datasets, as well as race/ethnicity and insurance type in the CXR dataset specifically.

Additionally, we measure the FNR for the no finding label across all subgroups (the definitions are similar to equation (1) and equation (2), but with $\hat Y = 0$ and *Y* = 1 with the patients belonging to *s*_*j*_ or *s*_*i,j*_). This measure is useful to help differentiate between overall model noise (for example, when predictions are flipped at random in either direction), which would result in approximately correlated FPR and FNR rates across subgroups, and selective model noise (for example, when predictions are selectively biased towards a prediction of no finding), which would result in un- or anti-correlated FPR and FNR rates. Although both kinds of noise are problematic, the latter is a form of technical bias amplification because it would show the known bias of clinical underdiagnosis as being selectively amplified by the algorithm—that is, the model is not only failing to diagnose those patients who clinicians are misdiagnosing, but it may also fail to diagnose other patients who clinicians did not underdiagnose.

Finally, we evaluate the FDR for the no finding label across all subgroups, defined in equation (3). FDR (or, equivalently, positive predictive value (PPV)) is a common metric used to evaluate the performance of classifiers. For our problem, this corresponds to the likelihood that a patient is ill given that the classifier predicts no finding.

3 $${\mathrm{FDR}}_{s_{i,j}} = P\left[ {Y = 0|s_{i,j},\,\hat Y = 1} \right]$$

### Medical images and labels preprocessing

In the CXR and CXP datasets the images are labeled with either a ‘positive’, ‘negative’, ‘uncertain’ or ‘not mentioned’ label. As in ref. ^7^, we aggregate all the non-positive labels to a negative label (that is, 0) and train the classifiers via multi-label classification, although we focus solely on the no finding label to examine underdiagnosis and the other fairness metrics. For each image, the no finding label is 1 if none of the disease labels are ‘positive’. All images are resized to 256 × 256 pixels following standard practice^7,16^ and are normalized using the mean and standard deviation of the ImageNet^42^ dataset.

### Model training

The trained models used in this study are identical to that of ref. ^7^ for all datasets, except for the NIH dataset. We train a 121-layer DenseNet^43^, with weights initialized using ImageNet^42^. Given that we need the no finding label, we include this label in the training of the model on the NIH dataset as well as all the other datasets. The train–validation–test set sizes for the ALL dataset are 575,381–67,177–65,068, for the CXR dataset they are 298,137–37,300–36,421, for the CXP dataset they are 178,352–23,022–22,274 and for the NIH dataset they are 98,892–6,855–6,373, respectively. The splits are random, and no patient is shared across splits. We use the same split as in ref. ^7^. The ALL dataset aggregates the original splits of the CXP, CXR and NIH datasets. Therefore, patients in the test set of each individual dataset stay in the test set of the ALL split. We applied center crop and random horizontal flip data augmentation. Similar to ref. ^7^, for the NIH dataset we applied a 10°, and for the other datasets we applied a 15° random rotation data augmentation for model training. Adam optimization with default parameters and binary cross-entropy loss functions are applied^7^. We have initialized the learning rate to 0.0005 and implement an early stop condition so that the learning rate drops to half if validation loss does not improve over three epochs, and the model stops training if no validation loss deduction occurs over 10 epochs.

All of the reported metrics such as the AUC, FPR, FNR and FDR are evaluated on the same test set. However, they are evaluated in each of five models (the same model trained five times with five different random seeds^7^), with the train–validation–test split kept fixed in the training of the five models. The seeds have been chosen randomly from numbers between 0 and 100. Thus, per dataset, the reported outcomes—that is, the AUC, FPR, FNR and FDR (Fig. 2, Extended Data Figs. 1–3 and Supplementary Table 7)—in this study are the average of the outcomes of the five models (with different random seed initializations) ± the 95% confidence interval . Following best practice^16,32^ for FPR, FNR and FDR estimation, we select a single threshold for all groups, which maximizes the F1 score. Moreover, the protected attributes may not be available for all of the images. Only images that do not have missing corresponding values are considered in the count and in the FPR, FNR and FDR analysis. However, all of the images have been used for training the models, regardless of their protected attributes. Only medical images have been fed into the model at train and test times and the protected attributes of the patients have not been used in the model.

### Model performance

The average AUC of our models over all of the labels is given for each dataset in Table 1. To the best of our knowledge, our classifiers are either state of the art (SOTA) (14 labels for the CXP and CXR datasets and eight shared labels for the ALL dataset)^19–22^ or near SOTA (15 labels for NIH)^22^ in the multi-label disease classification task, as measured by AUCs averaged across all of the labels for each dataset. In Supplementary Table 7, our trained models are compared with the SOTA models. For the CXP dataset, the SOTA models^27^ and the leaderboard ranking (https://stanfordmlgroup.github.io/competitions/chexpert/) used a private, unreleased dataset of only 200 images^27^ and five labels, whereas we used a randomly sub-sampled test set of 22,274 images. Thus, our results are not directly comparable with those. Also, for the NIH dataset, the SOTA model^1^ is trained on 14 disease labels only, whereas we also included the label ‘no finding’ (15 labels).

### Reporting Summary

Further information on research design is available in the Nature Research Reporting Summary linked to this article.

## Online content

Any methods, additional references, Nature Research reporting summaries, source data, extended data, supplementary information, acknowledgements, peer review information; details of author contributions and competing interests; and statements of data and code availability are available at 10.1038/s41591-021-01595-0.

## Supplementary information

Supplementary Information Supplementary Tables 1–7

Reporting Summary

## Data Availability

All three datasets used for this work are public under data use agreements. We have followed all protocols associated with the data use agreements, and the experiments are conducted on observational, retrospective data. All datasets are referenced in the paper: the MIMIC-CXR^26^ dataset is available at https://physionet.org/content/mimic-cxr/2.0.0/, the CheXpert^27^ dataset is available at https://stanfordmlgroup.github.io/competitions/chexpert/ and the ChestX-ray14^28^ dataset is available at https://www.nih.gov/news-events/news-releases/nih-clinical-center-provides-one-largest-publicly-available-chest-x-ray-datasets-scientific-community. Access to all three datasets requires user registration and the signing of a data use agreement, after which access is provided in a timely manner. Only the MIMIC-CXR dataset requires the completion of an additional credentialing process. After following these procedures, the MIMIC-CXR data are available through PhysioNet^44^. The MIMIC-CXR project page on PhysioNet describes the data access procedure^45^. The race/ethnicity and insurance type for the patients are not provided directly with the download of the MIMIC-CXR dataset. However, these data are available by merging the patient IDs in MIMIC-CXR with subject IDs in MIMIC-IV^46^ using the patient and admissions tables. Access to MIMIC-IV requires a similar procedure as MIMIC-CXR and the same credentialing process is applicable for both datasets.

## Extended data

are available for this paper at 10.1038/s41591-021-01595-0.

## Code availability

The code for training the models on the MIMIC-CXR (CXR)^26^, CheXpert (CXP)^27^ and ALL datasets is identical to that in https://github.com/LalehSeyyed/CheXclusion. The code for training the ChestX-ray14 (NIH)^28^ dataset on 15 labels as well as the code for all of the analyses in this paper is presented in https://github.com/LalehSeyyed/Underdiagnosis_NatMed. We have provided the Conda environment in the same repository for the purpose of reproducibility. We are not able to share the trained model and the true labels and predicted labels CSV files of the test set due to the data-sharing agreement. However, we have provided the patient ID per test splits, random seed and the code. The true label and predicted label CSV files and trained models can then be generated by users who have downloaded the data from the original source following the procedure described in the Data Availability section.
