# Supplementary material for: Underdiagnosis bias of artificial intelligence algorithms applied to chest radiographs in under-served patient populations
Source: Nat Med. 2021 Dec 10;27(12):2176–82. doi: 10.1038/s41591-021-01595-0 (PMC8674135; doi:10.1038/s41591-021-01595-0)
Supplement: Supplementary file 1 — Supplementary Tables 1–7 [file 41591_2021_1595_MOESM1_ESM.pdf]

nature portfolio

https://doi.org/10.1038/s41591-021-01595-0

Supplementary information

Underdiagnosis bias of artificial intelligence algorithms applied to chest radiographs in under-served patient populations

In the format provided by the authors and unedited

Supplementary Materials for

Underdiagnosis bias of artificial intelligence algorithms applied to chest radiographs in under-served patient populations

Laleh Seyyed-Kalantari[1,2]*, Haoran Zhang[3], Matthew B. A. McDermott[3], Irene Y. Chen[3], Marzyeh Ghassemi[3]

[1]University of Toronto.

[2]Vector Institute.

[3]Massachusetts Institute of Technology.

*Correspondence to: laleh@cs.toronto.edu.

This PDF file includes:

Table. S1 to S7

**ALL**	0-20		20-40		40-60		60-80		80-	
No Finding	0	1	0	1	0	1	0	1	0	1
Male	377	333	3679	2679	8550	4000	10163	2514	2720	269
Female	198	481	2325	3011	6924	3951	7572	2065	2862	389
**CXR**	0-20		20-40		40-60		60-80		80-	
No Finding	0	1	0	1	0	1	0	1	0	1
Male	156	155	1838	1828	4371	2659	4876	1691	853	185
Female	89	397	1222	2374	3816	2770	4130	1526	1163	316
**CXP**	0-20		20-40		40-60		60-80		80-	
No Finding	0	1	0	1	0	1	0	1	0	1
Male	103	28	1476	351	3550	490	4864	377	1849	71
Female	82	-	861	244	2400	396	3126	247	1680	65
**NIH**	0-20		20-40		40-60		60-80		80-	
No Finding	0	1	0	1	0	1	0	1	0	1
Male	118	150	373	504	626	869	422	441	26	16
Female	27	70	234	389	711	767	317	297	-	-

Table S1 The ALL, CXR, CXP, and NIH test set, images count in the intersection of sex-age. The numbers for subgroup with too few members (<= 15) are omitted.

	0-20		20-40		40-60		60-80		80-	
**No Finding**	**0**	**1**	**0**	**1**	**0**	**1**	**0**	**1**	**0**	**1**
**Medicare**	39	-	263	312	2378	1232	4736	1339	1091	238
**Other**	85	194	1447	1468	3153	1947	2270	825	410	103
**Medicaid**	-	29	359	496	920	495	265	93	33	-
**White**	108	145	930	1043	4278	2156	5143	1532	1159	260
**Black**	19	46	617	783	1225	853	942	343	121	45
**Hispanic**	-	-	315	306	399	347	268	118	-	-
**Other**	-	-	70	64	230	157	364	119	55	-
**Asian**	-	19	64	50	115	107	160	89	66	-
**Native**	-	-	-	-	-	-	30	-	-	-

Table S2: The CXR test set, images count in the intersection of insurance-age and race/ethnicity-age subgroups. The numbers for subgroups with too few members (<= 15) are omitted.

	Medicare		Other		Medicaid		Male		Female	
**No Finding**	**0**	**1**	**0**	**1**	**0**	**1**	**0**	**1**	**0**	**1**
**Male**	4526	1556	4232	2097	757	473	-	-	-	-
**Female**	3981	1574	3133	2440	834	642	-	-	-	-
**White**	6283	2181	4701	2548	633	398	6688	2717	4930	2419
**Black**	1181	614	1276	1079	467	375	1152	659	1772	1411
**Hispanic**	230	126	461	435	307	221	547	308	451	474
**Other**	267	97	397	205	61	53	472	220	251	135
**Asian**	122	41	195	174	90	52	225	116	182	151
**Native**	30	-	-	-	-	-	23	-	18	-

Table S3: The CXR test set, images count in the intersection of sex-insurance, insurance-race/ethnicity, and sex-race/ethnicity. The numbers for the intersections with too few members (<= 15) are omitted (e.g. Native with label 1).

	White Male		White Female		Black Male		Black Female	
	FP No Finding	Not Healthy	FP No Finding	Not Healthy	FP No Finding	Not Healthy	FP No Finding	Not Healthy
**Airspace Opacity**	22.5% ± 0.8%	34.3%	26.1% ± 1.1%	34.2%	19.4% ± 2.0%	31.6%	20.3% ± 1.0%	29.3%
**Atelectasis**	15.6% ± 1.0%	28.5%	17.8% ± 0.8%	29.3%	15.9% ± 1.3%	27.3%	16.3% ± 0.6%	23.4%
**Cardiomegaly**	18.0% ± 1.1%	28.6%	16.8% ± 1.2%	28.0%	19.0% ± 0.5%	32.7%	29.6% ± 1.1%	37.9%
**Consolidation**	-	7.4%	-	6.5%	-	5.0%	-	5.7%
**Edema**	3.9% ± 0.1%	17.7%	3.8% ± 0.1%	19.8%	-	15.4%	-	15.7%
**Enlarged Cardiomediastinum**	3.6% ± 0.2%	5.1%	3.6% ± 0.6%	4.3%	-	3.6%	-	3.2%
**Fracture**	5.9% ± 0.4%	3.5%	6.4% ± 0.4%	3.6%	-	1.7%	-	1.0%
**Lung Lesion**	5.2% ± 0.6%	3.8%	6.1% ± 0.8%	4.6%	10.3% ± 0.7%	5.6%	5.8% ± 0.5%	4.9%
**Pleural Effusion**	5.5% ± 0.3%	35.0%	3.4% ± 0.4%	40.3%	-	19.4%	-	27.4%
**Pleural Other**	-	2.1%	-	1.2%	-	-	-	-
**Pneumonia**	7.6% ± 0.9%	10.4%	9.4% ± 0.6%	11.0%	9.1% ± 0.7%	10.6%	8.8% ± 0.9%	10.6%
**Pneumothorax**	2.3% ± 0.5%	6.2%	-	4.7%	-	2.8%	-	4.2%

Table S4: The prevalence of different diseases for the four intersections of race and sex in the CXR test set for the subset of the population who do not have a positive label for 'No Finding' ("Not healthy"), and the subset of the "Not Healthy" population who we falsely predict to have no finding (shown as a 95% confidence interval over 5 models with different random seeds). Note that points corresponding to tasks where the subgroup contains less than 15 positively labeled patients are omitted from the table.

	White Male	White Female	Black Male	Black Female
**FP No Finding**	1153 ± 63	823 ± 53	295 ± 11	512 ± 20
**Not Healthy**	6688	4930	1152	1772

Table S5: Sample sizes for the cohorts described in Table S4 from the test set of MIMIC-CXR. The "Not healthy" group corresponds to the number of samples with No Finding = 0, and is shown as a point estimate from the test set. The "FP No Finding" group is the subset of the "Not healthy" group which our model falsely predicts to have No Finding = 1, and is shown as a 95% confidence intervals obtained over 5 models with different random seeds, each with a different size for the underdiagnosed population.

Group	CXR	CXP	NIH	ALL
**Male**	33.6% ± 0.5%	56.7% ± 0.7%	29.8% ± 1.1%	34.8% ± 0.7%
**Female**	29.4% ± 0.4%	54.8% ± 0.8%	30.3% ± 1.2%	31.6% ± 0.6%
**80-**	44.2% ± 0.8%	67.9% ± 1.9%	53.3% ± 3.3%	46.8% ± 1.4%
**60-80**	40.4% ± 0.5%	63.7% ± 0.8%	34.9% ± 1.6%	41.1% ± 1.0%
**40-60**	33.0% ± 0.4%	55.2% ± 0.7%	30.3% ± 0.8%	34.4% ± 0.7%
**20-40**	23.3% ± 0.4%	49.1% ± 0.8%	26.6% ± 1.2%	26.1% ± 0.2%
**0-20**	18.1% ± 0.6%	50.0% ± 1.4%	22.1% ± 1.0%	20.2% ± 0.3%
**White**	34.0% ± 0.6%	N/A	N/A	N/A
**Native**	42.0% ± 4.7%	N/A	N/A	N/A
**Other**	31.0% ± 0.8%	N/A	N/A	N/A
**Asian**	27.6% ± 0.5%	N/A	N/A	N/A
**Hispanic**	29.5% ± 0.7%	N/A	N/A	N/A
**Black**	31.9% ± 0.3%	N/A	N/A	N/A
**Other**	29.1% ± 0.4%	N/A	N/A	N/A
**Medicaid**	31.9% ± 0.6%	N/A	N/A	N/A

Table S6: False discovery rates (FDR) for the “No Finding” label for MIMIC-CXR (CXR), CheXpert (CXP), and Chest-Xray14 (NIH) as well as the multi-source ALL dataset.

Dataset	Ours	*(19)*	*(20)*	*(21)*	*(22)*
**CXR**	0.834±0.001	0.830	0.830	0.83	--
**CXP**	0.805±0.001	--	--	0.80	0.807
**NIH**	0.835±0.002	--	--	--	0.87

Table S7: The comparison of our average AUC over all labels with the state of the art (SOTA) models.
