# Supplementary material for: Underdiagnosis bias of artificial intelligence algorithms applied to chest radiographs in under-served patient populations
Source: Nat Med. 2021 Dec 10;27(12):2176–82. doi: 10.1038/s41591-021-01595-0 (PMC8674135; doi:10.1038/s41591-021-01595-0)
Supplement: Supplementary file 2 — Reporting Summary [file 41591_2021_1595_MOESM2_ESM.pdf]

Corresponding author(s): Laleh Seyyed-Kalantari

Last updated by author(s): Oct. 22, 2021

Reporting Summary

Nature Research wishes to improve the reproducibility of the work that we publish. This form provides structure for consistency and transparency in reporting. For further information on Nature Research policies, see our Editorial Policies and the Editorial Policy Checklist.

Statistics

For all statistical analyses, confirm that the following items are present in the figure legend, table legend, main text, or Methods section.

n/a	Confirmed	
☐	☒	The exact sample size (*n*) for each experimental group/condition, given as a discrete number and unit of measurement
☐	☒	A statement on whether measurements were taken from distinct samples or whether the same sample was measured repeatedly
☒	☐	The statistical test(s) used AND whether they are one- or two-sided<br>*Only common tests should be described solely by name; describe more complex techniques in the Methods section.*
☒	☐	A description of all covariates tested
☒	☐	A description of any assumptions or corrections, such as tests of normality and adjustment for multiple comparisons
☐	☒	A full description of the statistical parameters including central tendency (e.g. means) or other basic estimates (e.g. regression coefficient) AND variation (e.g. standard deviation) or associated estimates of uncertainty (e.g. confidence intervals)
☒	☐	For null hypothesis testing, the test statistic (e.g. *F*, *t*, *r*) with confidence intervals, effect sizes, degrees of freedom and *P* value noted<br>*Give P values as exact values whenever suitable.*
☒	☐	For Bayesian analysis, information on the choice of priors and Markov chain Monte Carlo settings
☒	☐	For hierarchical and complex designs, identification of the appropriate level for tests and full reporting of outcomes
☒	☐	Estimates of effect sizes (e.g. Cohen's *d*, Pearson's *r*), indicating how they were calculated

Our web collection on statistics for biologists contains articles on many of the points above.

Software and code

Policy information about availability of computer code

Data collection

Data was publicly available. We have not collected the data and no software was used in our side to collect data. We have provided a full reference on data availability session.

Data analysis

The code for training the models on MIMIC-CXR (CXR) (26), CheXpert (CXP) (27), and ALL datasets is identical in (https://github.com/LalehSeyyed/CheXclusion). The code for training ChestX-ray14 (NIH) (28) datasets on 15 labels as well as the code for all the analyses in this paper is presented in (https://github.com/LalehSeyyed/Underdiagnosis_NatMed). We have provided the yml Conda environment in the same repository for reproducibility purposes. We are not able to share the trained model and the true label and predicted label CSV files of the test set due to the data-sharing agreement. But we have provided the patient ID per test splits, random seed, and the code. Then the true label and predicted label CSV files and trained models can be generated by users who have downloaded the data from the original source following the procedure that is described in 'Data availability' session.

```
channels:
  - pytorch
  - conda-forge
  - anaconda
  - defaults
dependencies:
  - blas=1.0=mkl
  - ca-certificates=2019.5.15=0
  - certifi=2019.3.9=py36_0
  - cffi=1.12.3=py36h2e261b9_0
  - cudatoolkit=10.0.130=0
  - cycler=0.10.0=py_1
```

```
- dbus=1.13.2=h714fa37_1
- expat=2.2.5=hf484d3e_1002
- fontconfig=2.13.1=he4413a7_1000
- freetype=2.9.1=h8a8886c_1
- gettext=0.19.8.1=hc5be6a0_1002
- glib=2.56.2=had28632_1001
- gst-plugins-base=1.14.0=hbbd80ab_1
- gstreamer=1.14.0=hb453b48_1
- icu=58.2=hf484d3e_1000
- intel-openmp=2019.4=243
- joblib=0.13.2=py36_0
- jpeg=9b=h024ee3a_2
- kiwisolver=1.1.0=py36hc9558a2_0
- libedit=3.1.20181209=hc058e9b_0
- libffi=3.2.1=hd88cf55_4
- libgcc-ng=8.2.0=hdf63c60_1
- libgfortran-ng=7.3.0=hdf63c60_0
- libiconv=1.15=h516909a_1005
- libpng=1.6.37=hbc83047_0
- libstdcxx-ng=8.2.0=hdf63c60_1
- libtiff=4.0.10=h2733197_2
- libuuid=2.32.1=h14c3975_1000
- libxcb=1.13=h14c3975_1002
- libxml2=2.9.9=h13577e0_0
- matplotlib=3.1.0=py36_1
- matplotlib-base=3.1.0=py36hfd891ef_1
- mkl=2019.4=243
- mkl-service=2.0.2=py36h7b6447c_0
- mkl_fft=1.0.12=py36ha843d7b_0
- mkl_random=1.0.2=py36hd81dba3_0
- ncurses=6.1=he6710b0_1
- ninja=1.9.0=py36hfd86e86_0
- numpy=1.16.4=py36h7e9f1db_0
- numpy-base=1.16.4=py36hde5b4d6_0
- olefile=0.46=py36_0
- openssl=1.1.1=h7b6447c_0
- pcre=8.43=he6710b0_0
- pip=19.1.1=py36_0
- pthread-stubs=0.4=h14c3975_1001
- pycparser=2.19=py36_0
- pyparsing=2.4.0=py_0
- pyqt=5.6.0=py36h13b7fb3_1008
- python=3.6.8=h0371630_0
- python-dateutil=2.8.0=py_0
- pytorch=1.1.0=py3.6_cuda10.0.130_cudnn7.5.1_0
- qt=5.6.3=h8bf5577_3
- readline=7.0=h7b6447c_5
- scikit-learn=0.21.2=py36hd81dba3_0
- setuptools=41.0.1=py36_0
- sip=4.18.1=py36hf484d3e_1000
- six=1.12.0=py36_0
- sqlite=3.28.0=h7b6447c_0
- tk=8.6.9=hed695b0_1002
- torchvision=0.3.0=py36_cu10.0.130_1
- tornado=6.0.2=py36h516909a_0
- tqdm=4.31.1=py36_1
- wheel=0.33.4=py36_0
- xorg-libxau=1.0.9=h14c3975_0
- xorg-libxdmcp=1.1.3=h516909a_0
- xz=5.2.4=h14c3975_4
- zlib=1.2.11=h7b6447c_3
- zstd=1.3.7=h0b5b093_0
- pip:
  - backcall==0.1.0
  - decorator==4.4.0
  - ipython==7.5.0
  - ipython-genutils==0.2.0
  - jedi==0.13.3
  - pandas==0.24.2
  - parso==0.4.0
  - pexpect==4.7.0
  - pickleshare==0.7.5
  - pillow==4.1.1
  - prompt-toolkit==2.0.9
  - ptyprocess==0.6.0
  - pygments==2.4.2
```

- pytz==2019.1
- scipy==0.18.1
- traitlets==4.3.2
- wcwidth==0.1.7

For manuscripts utilizing custom algorithms or software that are central to the research but not yet described in published literature, software must be made available to editors and reviewers. We strongly encourage code deposition in a community repository (e.g. GitHub). See the Nature Research guidelines for submitting code & software for further information.

Data

Policy information about availability of data

All manuscripts must include a data availability statement. This statement should provide the following information, where applicable:

- Accession codes, unique identifiers, or web links for publicly available datasets
- A list of figures that have associated raw data
- A description of any restrictions on data availability

All three datasets that we have used for this work are public and under data use agreements. We have followed the data use agreements, and the experiments are based on observational, retroactive data. The datasets are all well-referenced in the paper. Here is the link to each of the datasets:
MIMIC-CXR (26) dataset is available at: https://physionet.org/content/mimic-cxr/2.0.0/
CheXpert (27) dataset is available at: https://stanfordmlgroup.github.io/competitions/chexpert/
ChestX-ray14 (28) dataset is available at: https://www.nih.gov/news-events/news-releases/nih-clinical-center-provides-one-largest-publicly-available-chest-x-ray-datasets-scientific-community
Access to all three datasets requires user registration and the signing of a data use agreement. Then access is provided in a timely manner. Only the MIMIC-CXR dataset also requires the completion of a credentialing process, that takes a few hours to be completed. After following this procedure the MIMIC-CXR data is available through PhysioNet (42). The MIMIC-CXR project page on PhysioNet describes the data access procedure (43). The race/ethnicities and insurance type of the patients are not provided naturally with the download of the MIMIC-CXR dataset. However, this data is available through merging the patient IDs in MIMIC-CXR with subject IDs in MIMIC-IV (44) using the patient and admissions tables. Access to MIMIC-IV requires a similar procedure as MIMIC-CXR and the same credentialing process is applicable for access to both datasets.

Field-specific reporting

Please select the one below that is the best fit for your research. If you are not sure, read the appropriate sections before making your selection.

☒ Life sciences ☐ Behavioural & social sciences ☐ Ecological, evolutionary & environmental sciences

For a reference copy of the document with all sections, see nature.com/documents/nr-reporting-summary-flat.pdf

Life sciences study design

All studies must disclose on these points even when the disclosure is negative.

Sample size	In total, we use over 98,000 chest x-ray images for model training, with specific ablations for subgroups and dataset source. While there is no definitive threshold set for convergence of deep neural network model training, the machine learning literature generally suggests that over 50,000 samples is appropriate for convolutional neural network convergence in fine-tuning of natural and medical images. We have randomly samples 10% , 10%, and 80% of the patients of each whole dataset for train, validation and test set, such that each patient medical images belongs to only one of the train, test and validation sets. We have not done any other sampling form the whole dataset in our model development. The detail on the number of images per dataset (sample size) and where they have been collected are presented in Human research participant session in this document (next page.)
Data exclusions	No data was excluded.
Replication	To ensure reproducibility, we save all model random seeds, and have released the source code for model training upon acceptance.
Randomization	As no human subject evaluation was performed, we did not require randomization groups.
Blinding	As this study contained no human evaluation or intervention, and only profiles computational models on a fixed dataset, no blinding was necessary

Reporting for specific materials, systems and methods

We require information from authors about some types of materials, experimental systems and methods used in many studies. Here, indicate whether each material, system or method listed is relevant to your study. If you are not sure if a list item applies to your research, read the appropriate section before selecting a response.

Materials & experimental systems

n/a	Involved in the study
☒	☐ Antibodies
☒	☐ Eukaryotic cell lines
☒	☐ Palaeontology and archaeology
☒	☐ Animals and other organisms
☐	☒ Human research participants
☒	☐ Clinical data
☒	☐ Dual use research of concern

Methods

n/a	Involved in the study
☒	☐ ChIP-seq
☒	☐ Flow cytometry
☒	☐ MRI-based neuroimaging

Human research participants

Policy information about studies involving human research participants

Population characteristics

We have used already existing public data on human Chest X-rays and we have not collect them. The distribution og data over sex and age is provided in table 2. One one of the datasets has the race and insurance type of the patients where we have reported in the Table 2.

#images
CXR: 371,858
CXP: 223,648
NIH: 112,120

Sex
CXR -Male: 52.17%
CXR- Femle: 47.83%
CXP -Male: 59.36%
CXP -Female: 40.64%
NIH-Male: 56.49%
NIH- Female: 43.51%

Age - CXR
0-20 2.20%
20-40 19.51%
40-60 37.20%
60-80 34.12%
80+ 6.96%

Age - CXP
0-20 0.87%
20-40 13.18%
40-60 31.00%
60-80 38.94%
80+ 16.01%

Age - NIH
0-20 6.09%
20-40 25.96%
40-60 43.83%
60-80 38.94%
80+ 1.01%

CXR: Race/Ethnicity
Asian 3.24%
Black 18.59%
Hispanic 6.41%
Native 0.29%
White 67.64%
Other 3.83%
--
CXR - Insurance
Medicare 46.07%
Medicaid 8.98%
Other 44.95%

Recruitment

Because of the size of these large datasets, and the fact that no exclusionary criteria are mentioned in the dataset descriptions, we do not anticipate any issues with selection bias and assume that the collected datasets are representative of patients at these hospitals over the specified years. Only the ChestX-ray14 dataset is gathered from the NIH clinical research dedicated hospital where patients “are treated without charge, unlike most hospitals, the Clinical Center does not routinely

provide standard diagnostic and treatment services. Admission is selective: patients are chosen by Institute physicians solely because they have an illness being studied by those Institutes", as mentioned in their website (https://clinicalcenter.nih.gov/about/welcome/faq.html)
NIH dataset has only frontal view images where the other datasets have both frontal and lateral view images. We include all the images of each dataset regardless of their view in the model training and evaluation.
The race/ethnicity and sex data are self-reported in the MIMIC-CXR dataset and age is reported at a patient's first admission. In the CheXpert dataset, sex is assigned by clinicians and the age is at the time of the examination. In the ChestX-ray14 dataset, the sex is self-identified and the age corresponds to the time of the examination. In the MIMIC-CXR dataset, we only have the race/ethnicity and insurance type data of a patient if the patient was admitted to an ICU, so there are around ~100,000 x-rays where we do not have this data (these are x-rays done for patients who were only admitted to the emergency department. The reported races/ethnicities in MIMIC-CXR dataset are WHITE, OTHER, HISPANIC/LATINO, BLACK/AFRICAN AMERICAN, AMERICAN INDIAN/ALASKA NATIVE, where in this study we have used shorter terminology White, Other, Hispanic, Black, and Native for each group, respectively.

Ethics oversight

Since we have worked on public, anonymized, retrospectively collected data, and we have not collect any human data ourselve we did not get any organizational/IRB approval. These public datasets are largely and commonly used in the machine learning medical imaging literature.

Note that full information on the approval of the study protocol must also be provided in the manuscript.
